# Proteomic and microbiota analyses of the oral cavity during psychological stress

**DOI:** 10.1371/journal.pone.0268155

**Published:** 2022-05-25

**Authors:** Durga Paudel, Yasuhiro Kuramitsu, Osamu Uehara, Tetsuro Morikawa, Koki Yoshida, Sarita Giri, Syed Taufiqul Islam, Takao Kitagawa, Tadashi Kondo, Kazuki Sasaki, Hirofumi Matsuoka, Hiroko Miura, Yoshihiro Abiko

**Affiliations:** 1 Division of Oral Medicine and Pathology, School of Dentistry, Health Sciences University of Hokkaido, Hokkaido, Japan; 2 Advanced Research Promotion Center, Health Sciences University of Hokkaido, Hokkaido, Japan; 3 Division of Molecular Epidemiology and Disease Control, School of Dentistry, Health Sciences University of Hokkaido, Hokkaido, Japan; 4 Division of Periodontology and Endodontology, School of Dentistry, Health Sciences University of Hokkaido, Hokkaido, Japan; 5 Division of Pediatric Dentistry, School of Dentistry, Health Sciences University of Hokkaido, Hokkaido, Japan; 6 Division of Rare Cancer Research, National Cancer Center Research Institute, Tokyo, Japan; 7 Department of Peptidomics, Sasaki Institute, Tokyo, Japan; Pacific Northwest National Laboratory, UNITED STATES

## Abstract

Psychological stress is associated with various oral diseases such as aphthous stomatitis, oral lichen planus, taste disturbances and glossodynia. However, the underlying mechanism is still unknown. The aim of this study was to determine the effect of psychological stress on salivary proteins and the oral microbiota in a rat model of chronic restraint stress. Six-week-old Sprague Dawley rats were subjected to restraint stress for four hours daily for 1 month. The behavior, weights of the adrenal glands, and serum corticosterone levels were evaluated as stress markers. Proteomic analysis of the saliva was performed using two-dimensional gel electrophoresis followed by mass spectrometry and Western blotting. Analysis of the oral microbiota was performed via 16S rRNA next-generation sequencing. The low mean body weights, lower number of entries and time spent in the open arm of elevated plus maze, high adrenal gland/body weight ratios, and high serum corticosterone levels confirmed the high levels of stress in the stress group of rats compared to the controls. Thirty-three protein spots were found to be significantly altered between the two groups. After silver staining, seven visible spots were subjected for mass spectrometry, and the expression levels of the two most significantly altered proteins, BPI fold containing family A member 2 and von Ebner’s gland protein, were confirmed by Western blotting. 16S rRNA sequencing analysis revealed a significant reduction in alpha diversity in the stress group compared to the controls. The abundances of oral bacteria, such as *Facklamia* and *Corynebacterium*, were significantly altered between the two groups. Additionally, analysis with PICRUSt2 software predicted 37 different functional pathways to be altered between the groups. In conclusion, the present study identified altered salivary proteins and oral microbiota due to psychological stress. These findings might aid in understanding the pathogenesis of stress-related oral diseases.

## 1. Introduction

Several oral diseases, such as recurrent aphthous stomatitis (RAS), oral lichen planus, and conditions such as taste disturbances and glossodynia, might present with an unestablished etiology [1]. The involvement of multiple factors makes it difficult to understand the underlying pathogenesis of these diseases. Psychological stress might be one of the important factors [2,3]; however, its role in the etiology of these diseases remains unclear. Alterations in the oral environment, including the saliva and the oral microbiota, caused by psychological stress could affect the etiologies of these diseases.

Salivary proteins have been correlated with various oral diseases, such as chronic periodontitis and dental caries [3]. Although salivary proteins and hormones such as alpha-amylase, cortisol, chromogranin A, and immunoglobulin A have been shown as stress biomarkers [4,5], their role in the etiology of stress-related oral diseases remains unclear. The expression levels of inflammatory cytokines, including interleukins, tumor necrotic factor- α, and interferon- γ, in the saliva of patients with stress-related oral diseases have been reported previously; however, the levels have been found to vary among studies [6–8]. This might be attributed to the multiple local and systemic factors in the oral cavity that vary among individuals. Moreover, differences in sampling sites and methods among studies also contributes to varied results. The proteomic analysis of salivary proteins under psychological stress conditions using an animal model might help to understand the pathogenesis of stress-related oral diseases. Moreover, the altered proteins under psychological stress condition could be a novel salivary biomarker for psychological stress.

The oral cavity harbors second-largest and diverse microbiota after the gut [9]. Alterations in the oral microbiota can cause or be an indicator of various oral diseases including chronic periodontitis and dental caries [9]. The microbiota in the oral cavity can be affected by multiple local and systemic factors. Systemic diseases such as diabetes, gastrointestinal diseases, and liver diseases alter the oral microbiota [9,10]. Oral microbial biomarkers have been studied in various oral and systemic diseases such as periodontal diseases, oral cancer, pancreatic cancer, chronic pancreatitis and obesity [9,11–13]. Also, a study reported the impact of cortisol, a stress hormone on the metatranscriptome of the oral microbiome [14]. However, studies on the direct effect of psychological stress on the oral microbiota are limited. The identification of altered microbiota under psychological stress using an animal model could clarify its role in stress related oral diseases and also serve as a potential biomarker.

The purpose of the present study was to examine the alterations in the salivary proteins and the oral microbiota via proteomic and 16S rRNA sequencing analyses, respectively, in a rat model of chronic stress.

## 2. Materials and methods

### 2.1 Induction of psychological stress

Six-week-old male Sprague Dawley rats (Sankyo Labo, Sapporo, Japan) (n = 20) were divided into a control group and stress group (n  =  10, each group) with simple randomization. The sample size was determined based on the law of diminishing return using the resource equation method based on the law of diminishing return using the resource equation method [15]. The rats were housed in a cage (n = 2 per cage) at a room temperature of 25°C and 12/12h light-dark cycle. The rats had free access to standard rodent chow diet and water. Both group rats were acclimatized for 1 week to adapt to the environment. Stress was induced in the rats belonging to the stress group, according to an established protocol [16]. Briefly, the rats were enclosed in a ventilated plastic tube that was just sufficient to fit the rat in, thereby limiting free movement (S1A Fig). The protocol was continued for 4 h daily over a period of 1 month by the same researcher. The control group rats were left undisturbed in their cage for the entire duration except that they were handled once a week for body weight measurement. This study was approved by the animal ethics committee of the Health Sciences University of Hokkaido (Approval no: 19–084) and complied with the Animal Research: Reporting of In Vivo Experiments (ARRIVE) guidelines. The sample collection was performed under intraperitoneal injection of sodium pentobarbital (50 mg/kg) anesthesia with all effort for minimal suffering.

### 2.2 Sample collection

Specimens of the oral microbiota were collected using an oral swab (Isohelix, Cell Projects, Kent, UK). The oral cavity was swabbed for 30 s, starting from the dorsum of the tongue to the palate, buccal mucosa, upper and lower vestibules, and the floor of the mouth. The swab was then placed in 1.5 ml tubes containing 200 μl of Tris-EDTA buffer. Pilocarpine (5 mg/kg; Fujifilm, Osaka, Japan) was injected intraperitoneally to stimulate the saliva, which was then collected using a pipette. Serum was collected from blood withdrawn from the heart. The right adrenal gland was removed and weighed. The intestinal stool was collected from the terminal part of the large intestine. Oral microbial DNA was extracted following an established protocol [17] using the DNeasy Blood and Tissue kit (Qiagen, Hilden, Germany). Briefly, the oral swab was mixed with 200 μl of lysozyme (20 mg/ml, Fujifilm Wako Pure Chemicals, Japan) and incubated at 37°C for 60 min with moderate shaking. Proteinase K (25 μl) and Buffer AL (200 μl), obtained from the DNeasy Blood and Tissue kit (Qiagen, Germany), were added to the mix; the mixture was vortexed and incubated overnight at 56°C with moderate shaking. The swab was drained and then discarded. Next, 200 μl of 99% ethanol was added and vortexed following which, the contents were transferred to spin column from the DNeasy Blood and Tissue kit (Qiagen). The bind DNA to the column membrane was washed with wash buffer from the same kit (500 μl Buffer AW1 and AW2, each). The DNA was then eluted using 50 μl Buffer AE (Qiagen). Intestinal microbial DNA was collected using the DNAeasy PowerSoil Kit (Qiagen, Hilden, Germany), according to the manufacturer’s protocol.

### 2.3 Stress markers

The following stress markers were evaluated and a single assessor for each stress marker blinded to the group allocation performed the experiment: i) The body weight of each rat was recorded every week. ii) The right adrenal glands were excised and the weight of the gland per body weight was calculated. iii) The serum corticosterone level was evaluated using the Enzyme-Linked Immunosorbent Assay kit (Arbor Assays, Michigan, USA) iv) The behavior of the rat was analyzed using the elevated plus-maze test (EPMT) after 1 month of the stress protocol using custom-made maze of standard dimensions (S1B Fig) [18]. The rats were placed at the center of the maze facing the open arm and allowed to explore it for 10 min. The number of entries and the time spent in the open and closed arms by each rat were recorded manually. The placing of rats in the maze and the recording of behavior was done by two different investigators and both were blinded to the groups.

### 2.4 Proteomic analysis of the salivary proteins

#### 2.4.1 Two-dimensional gel electrophoresis (2-DE)

The saliva was concentrated using Microcon centrifugal filter unit (YM-3; Sigma Aldrich, Germany) and protein concertation was measured using Lowry’s method. Then, 120 μg of protein from each sample was prepared using the ReadyPrep 2-D Cleanup kit (Bio-Rad, Hercules, CA USA). The protein pellet was resuspended in rehydration buffer (8M urea, 2% 3-Cholamidopropyl dimethylammonio-1-propanesulfonate, 0.01% bromophenol blue, 1.2% Destreak reagent, and 0.5% IPG buffer). The resuspended pellet was used for isoelectric focusing (IEF), which was performed using an IPGphor 3 IEF unit (GE Healthcare, Buckinghamshire, UK) on immobilized, linear-pH gradient strips (size, 11 cm; pH, 3–10; Bio-Rad). The following voltage program was used: rehydration for 10 h at 20°C (no voltage); stepwise increase from 0 V to 500 V for 4 h; 500 V to 1000 V for 1 h; 1000 V to 8000 V for 4 h; linear increase from 8000 V for 20 min; and a final phase of 500 V from 20,000 to 30,000 Vh. After IEF, the strips were reduced in a reducing buffer containing 50 mM Tris, 6 M urea, 30% glycerol, 2% sodium dodecyl sulfate (SDS), and 2% 2-mercaptoethanol for 10 min. It was then alkylated in an alkylation buffer containing 50 mM Tris, 6 M urea, 30% glycerol, 2% SDS, and 2.5% iodoacetamide. Sodium dodecyl sulfate-polyacrylamide gel electrophoresis (SDS-PAGE) was performed on a precast polyacrylamide gel (4–20% Criterion TGX Precast Gel # 5671091J10, BioRad, CA, US) at 200V for 30–40 min. After SDS-PAGE, the gels were fixed with a fixing solution containing 40% ethanol and 10% acetic acid for 2 h. The fixed gels were then stained with Flamigo Fluorescent Gel Stain (Bio-Rad) overnight and away from light. The gels then were washed with deionized water and subjected to image analysis. Protein spots in the gel were recorded using the LuminoGraph I and ImageSaver 6 software (ATTO Corporation, Tokyo, Japan). The expression levels of the protein were quantified based on the intensities of each spot using the Progenesis SameSpot software (Nonlinear Dynamics, Newcastle upon Tyne, UK).

#### 2.4.2. Mass spectrometry

After the image analysis, the gels were further stained with silver stain (ATTO, Tokyo, Japan) to visualize the spots following the manufacturer’s protocol. Briefly, the gels were fixed for 10 min, stained with staining solution for 5 min, developed until gels were stained then stop solution added after the gels were stained. The significantly altered spots that were visible after silver staining were picked for mass spectrometry. The silver stain was removed from the gel piece by rinsing with 15 mM potassium ferricyanide/50 mM sodium thiosulfate solution. The sample was then reduced in 50% acetonitrile/50mM ammonium bicarbonate/5 mM DTT solution, dehydrated in 100% acetonitrile, and then rehydrated with an in-gel digestion reagent containing 10 μg/mL sequencing-grade-modified trypsin (Promega, Madison, WI, USA) in 30% acetonitrile /50 mM ammonium bicarbonate/5 mM DTT overnight at 30˚C. The samples were lyophilized overnight by using Labconco77400 (Labconco, Kansas, MO, USA). Lyophilized samples were dissolved in 0.1% formic acid. A one-tenth volume of each sample was analyzed by liquid chromatography (LC) with a nanoLC ADVANCE UHPLC system (Michrom Bioresources, CA) connected to an Orbitrap XL mass spectrometer (Themofisher Scientific, CA). The analyte was separated on an L-column 2 (ODS, 3 um particle size, 0.1 x 150 mm, CERI, Japan). A linear ACN gradient of 5% to 40% over 100 min was applied with a flow rate of 250 nL/min. The mass spectrometer was operated in data dependent mode with ten most intense ions subjected to MS/MS. Full scan MS spectra (m/z 400–1300) were acquired in the Orbitrap with a resolution of 60,000, and MS/MS spectra in the ion trap mode. MS/MS spectra were interrogated against the NCBI NR rat database (September 2015, 84,189 sequences) using the identification software Mascot (version 2.4, Matrix Science, UK), with trypsin specified and up to two missed cleavages allowed. Precursor mass tolerance was 5 ppm and product ion mass tolerance 0.6 Da. Carbamidomethylcysteine was set as a fixed modification, and methionine oxidation and N-terminal acetylation as variable modifications.

#### 2.4.3 Western blotting

The candidate proteins were confirmed by Western blotting. SDS-PAGE was performed using 20–30 μg of the salivary proteins in precast AnyKD Mini-PROTEAN TGX gels (Bio-Rad). Subsequently, the proteins were transferred from the gels onto Immobilon -P Polyvinylidene fluoride (PVDF) membranes (Millipore, Billerica, MA, USA) with a semi-dry blotting system (Bio-Rad). The membranes were stained with 20 mL of 1% Ponceau S stain for 10 min for total protein normalization. Images were obtained and the gels were de-stained with Tris-buffered saline containing 0.05% Tween-20 (TBST) solution with pH 7.6. The membrane was then blocked with TBST containing 5% skimmed milk for 1 h and incubated with the following primary antibodies: monoclonal antibody against Splunc2 (BPIFA2) (Clone 1F12; 1:500 dilution, ThermoFisher Scientific, Waltham, MA, USA) and polyclonal antibody against Lipocalin 1 ((#MBS2027781; 2 μg/mL, MyBioSource Inc., San Diego, CA, USA) overnight at 4°C. The membranes were then incubated with HRP-conjugated secondary antibody (1:10,000; Jackson Immuno-Research Laboratories Inc, USA) for 1 h at room temperature. The protein bands were visualized using the enhanced chemiluminescence system Clarity Max Western ECL substrate (Bio-Rad) and LuminoGraph I (ATTO Corporation) and recorded using the ImageSaver6 software (ATTO Corporation). The bands were quantified using an image analysis software CS Analyzer 4 (ATTO Corporation).

### 2.5 Oral and intestinal microbiota analyses

The 16S metagenomic sequencing library preparation protocol (Illumina, San Diego, USA) was used to prepare the sequencing libraries. In brief, the following steps were followed: In the first step PCR (Amplicon PCR), the V3-V4 region primers (Amplicon Primers) were used to amplify the V3-V4 regions of the bacterial 16S ribosomal RNA (rRNA). A mixture of 2.5μl of microbial DNA, 5μl of Amplicon PCR forward and reverse primers, and 12.5μl of KAPA HiFi HS ReadyMix (Nippon Genetics, Tokyo, Japan) was prepared and PCR was performed in a thermal cycler under the following conditions: 95°C for 3 min, 25 cycles of 95°C for 30 s, 55°C for 30 s, 72°C for 30 s each followed by 72°C for 5 min, and hold at 4°C. PCR clean-up was done using AMPure XP beads (Beckman Coulter Life Sciences, Germany). In the second step PCR (Index PCR), KAPA HiFi HS ReadyMix and Nextera XT index kit (Illumina, USA) were used to attach dual indices and the Illumina sequencing adapters. Next, 5μl of PCR clean-up done DNA, Nextra XT index primer 1, and Nextra XT index primer 2 were mixed with 10 μl of PCR grade water. The PCR was performed on a thermal cycler under the following conditions: 95°C for 3 min, 8 cycles of 95°C for 30 s, 55°C for 30 s, 72°C for 30 s each followed by 72°C for 5 min, and hold at 4°C. PCR clean-up was done using AMPure XP beads (Beckman Coulter Life Sciences, California, USA). Library quantification was done using a Qubit 3 fluorometer; subsequently, the library was normalized and pooled as a 4 nM library. The pooled library was denatured with freshly prepared 0.2N NaOH and mixed with PhiX Control v3 (Illumina). Heat denaturation was performed at 96°C following which, the samples were loaded onto the Illumina MiSeq System for sequencing.

Data from the 16S rRNA sequencing were analyzed using Quantitative Insights into Microbial Ecology2 (QIIME2 v2020.4.0) against the 16S rRNA database (Greengenes v13.8). Sequence reads were demultiplexed using qiime tools import. The reads were further denoised and the feature selection was done using Amplicon sequence variant (ASV) approach in DADA2 pipeline. Diversities in microbiomes between the stress and control groups were analyzed as alpha diversity, beta diversity and taxonomic abundance. For alpha diversity, observed operational taxonomic units (OTUs), faith phylogenetic diversity and Shannon index were evaluated. The rarefaction curve was evaluated for the sequencing depth. For beta diversity, unweighted and weighted UniFrac distance metric based on three-dimensional principal coordinate analysis (PCoA) scatterplots were evaluated. Linear discriminant analysis (LDA) Effect Size (LefSe) was used to determine the significant differences in the taxa abundance between the two groups. Phylogenetic Investigation of Communities by Reconstruction of Unobserved States 2 (PICRUSt 2) was used to predict the metagenome function in Galaxy/ Hutlab (https://huttenhower.sph.harvard.edu/galaxy). The data of 16S rRNA sequencing is publicly available at National Center for Biotechnology Information Sequence Read Archive under BioProject PRJNA814321.

### 2.6 Statistical analysis

For the proteomic analysis, SPSS v 26.0 (IBM, USA) was used to analyze the data, and a p < 0.05 was considered as statistically significant. The significant differences in stress markers between the two groups were determined using Mann-Whitney U test. Assuming normal distribution, one-way analysis of variance (ANOVA) was used to determine the significant differences in intensities of protein spots. p < 0.05 was considered as statistically significant and uncorrected p values was used in all analyses. For the 16S rRNA data analysis, the Kruskal-Wallis test was used to test the significance of the alpha diversity. Permutational multivariate ANOVA (PERMANOVA) was used to test the significant differences in beta diversity evaluated as unweighted and weighted UniFrac distance matrix. The Welch’s t-test in Statistical Analysis of Metagenomic (and other) Profiles (STAMP) software was used to test the significant differences in the predicted metagenome function with 95% confidence interval. The adjusted p-value was calculated using Benjamini–Hochberg false discovery rate and p<0.05 was considered as statistically significant.

## 3 Results

### 3.1 Stress markers

**Fig 1:**
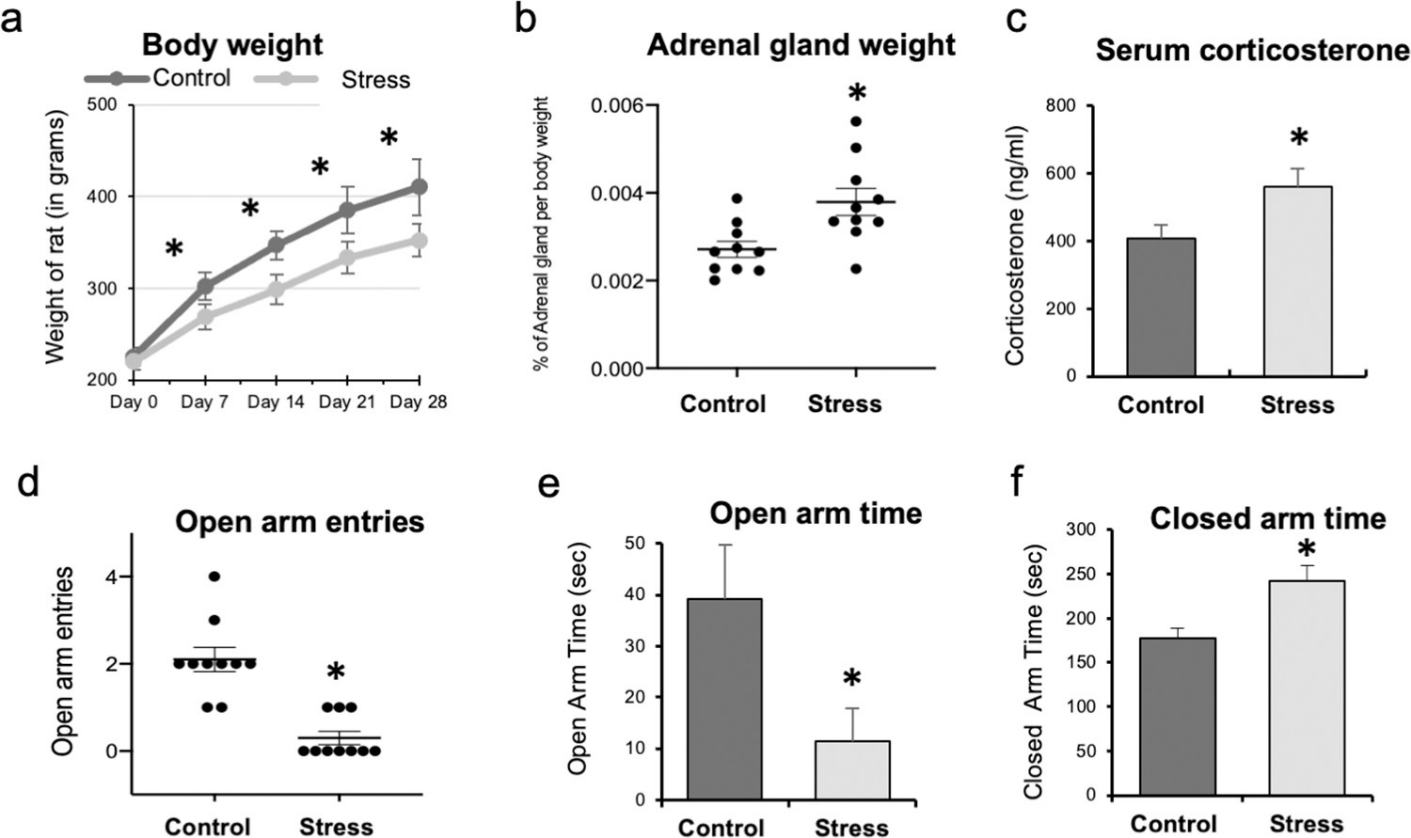
The results of the different stress markers. (a) From the first to fourth week of the experiments, the mean body weights of the rats in the stress group were significantly lower than those in the control group when compared at same time point (*p < 0.001; Mann Whitney-U test). (b) The mean weight of the adrenal gland per body weight were significantly higher in the stress group than in the control group (*p = 0.005; Mann Whitney-U test). (c) The serum corticosterone level after 1 month of the chronic stress protocol was significantly higher in the stress group compared to that in the control group (*p < 0.001; Mann Whitney-U test). (d, e, f) The graphs show the results of behavior analysis of rats using elevated plus maze test. The rats were placed at the center of the maze facing the open arm and allowed to explore it for 10 min. The number of entries and the time spent in the open and closed arms by each rat were recorded manually. (d, e) The number of open arm entries and the time spent in the open arm were significantly lower in the stress group compared to those in the control group (*p<0.001 and *p = 0.01, respectively; Mann Whitney-U test). (f) The time spent in the closed arm was significantly higher in the stress group as compared to that in the control group (*p = 0.007; Mann Whitney-U test).

The mean body weights of the animals in the stress groups were significantly lower during the first, second, third, and fourth weeks of the experiment as compared to the control group (p < 0.001; Fig 1A). The mean weight of adrenal gland per body weight was higher in the stress group as compared to the control group (p = 0.005; Fig 1B). The serum corticosterone level was significantly higher in the stress group after 1 month of chronic stress when compared to that in the control group (p < 0.001; Fig 1C). The EPMT showed that the number of open arm entries and the time spent in the open arm were significantly lower in the stress group compared to the control group (p < 0.001 and p = 0.01, respectively; Fig 1D and 1E). The time spent in the closed arm was significantly higher in the stress group compared to the control group (p = 0.007; Fig 1F). These data indicated that 1 month of the chronic restraint stress protocol induced significantly higher levels of stress in the stress group compared to that in the control group.

Changes in the intestinal microbiota caused by psychological stress (referred to as the “Brain-gut interaction”) are well documented [19]. Most of the studies showed a significant reduction in bacteria, such as *Lactobacillus* and *Bifidobacterium*, at the genus level under different stress conditions [19]. In the present study, the relative abundance of both *Lactobacillus* and *Bifidobacterium* was reduced in the stressed rats, thus supporting the stress protocol used (S2A and S2B Fig). Consistent with previous studies [20,21], significant differences in the beta diversity of the intestinal microbiota were noted between the stress and control groups (S3A and S3B Fig). Taken together, these findings in the intestinal microbiota suggest that our model is a reliable psychological stress animal model.

### 3.2 Proteomic analysis of the salivary proteins

**Fig 2:**
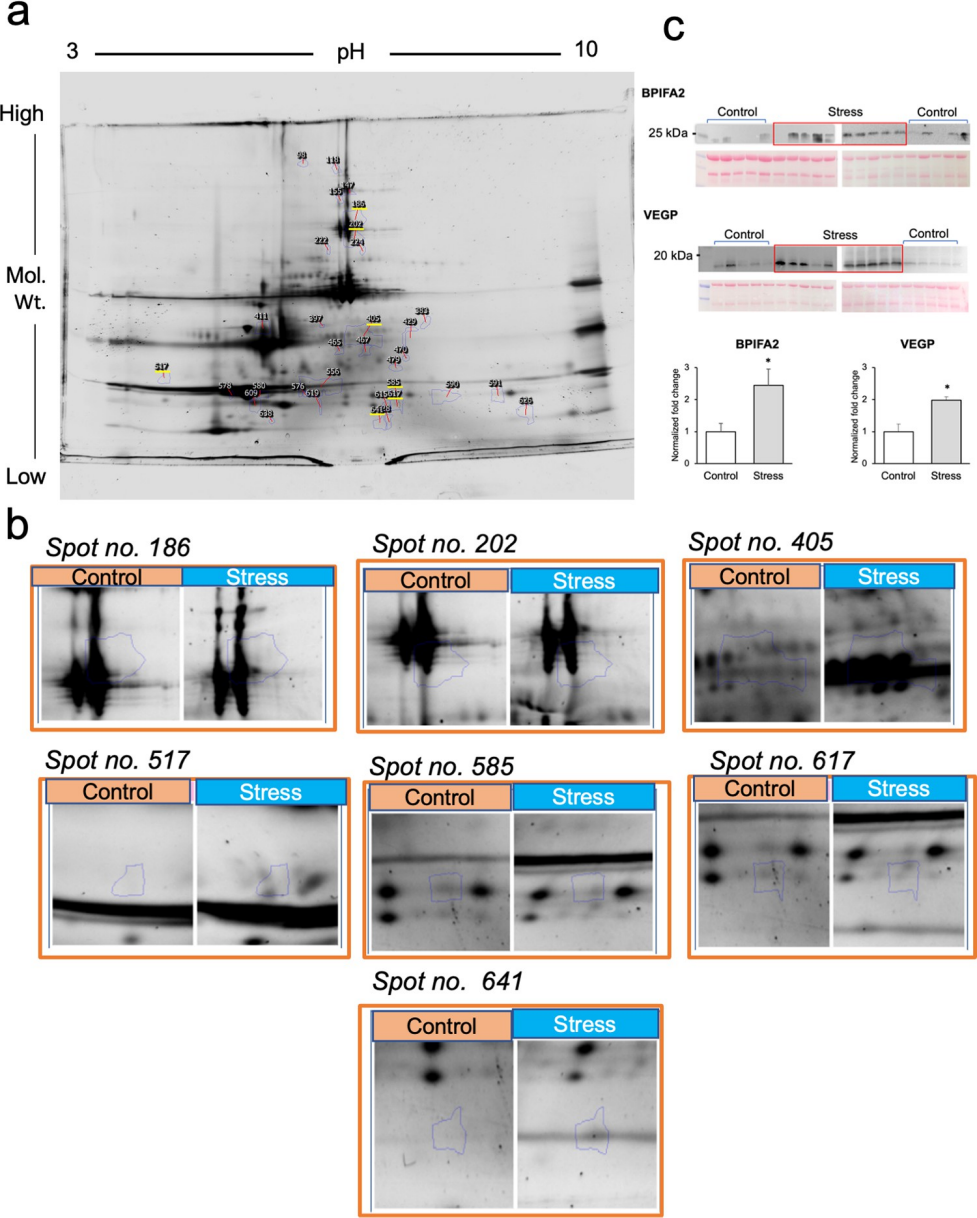
Identification of significantly altered protein spots in the gel and validation by Western blot analysis. The protein spots in each gel after fluorescence staining were identified using the LuminoGraph I and ImageSaver 6 software and quantified with the Progenesis SameSpot software. Eight gels from each group were used for quantification. (a) A representative gel image showing the protein spots. The spots underlined in yellow were visible after silver staining and were selected for mass spectrometry. (b) The magnified image of the protein spots was subjected to mass spectrometry. (c) The levels of two candidate proteins, BPI fold-containing family A member 2 (BPIFA2) and von Ebner’s gland protein (VEGP) were confirmed in all samples via Western blotting. The mean intensities of the bands were significantly higher in the stress group compared to those in the controls (*p < 0.05; Mann Whitney-U test).

The fluorescence-stained gel pictures were quantified to identify the significantly altered protein spots between the two groups (S4 Fig). The Progenesis SameSpot software identified 33 significantly altered spots between the control and stress groups (p < 0.05; Fig 2A). The average normalized values, fold changes and p-values of each spot are shown in Table 1.

**Table 1:** The significantly altered 33 proteins between stress group and control.

Spot No.	Average Normalized Volumes		Fold change	ANOVA (p)
	*Control*	*Stress*		
**98**	3.174E+005	1.477E+005	2.1	0.015
**118**	6.176E+005	3.174E+005	1.9	0.030
**147**	4.279E+006	1.531E+006	2.8	0.014
**155**	8.973E+005	1.895E+006	2.1	0.043
**186**	3.281E+007	1.793E+007	1.8	0.016
**202**	2.914E+007	1.955E+007	1.5	0.046
**222**	3.025E+005	5.700E+005	1.9	0.030
**224**	1.932E+005	3.991E+005	2.1	0.029
**383**	3.288E+005	4.722E+005	1.4	0.031
**397**	1.050E+005	2.096E+005	2.0	0.030
**405**	2.568E+007	4.097E+007	1.6	0.002
**411**	2.427E+007	1.717E+007	1.4	0.014
**429**	1.014E+006	1.612E+06	1.6	0.021
**465**	5.479E+005	1.063E+006	1.9	0.045
**467**	8.739E+005	2.445E+006	2.8	0.005
**470**	2.067E+005	3.274E+005	1.6	0.034
**479**	3.608E+005	6.077E+005	1.7	0.005
**517**	7.447E+005	1.904E+006	2.6	0.022
**556**	3.864E+007	5.441E+007	1.4	0.034
**576**	4.595E+005	1.261E+006	2.7	0.003
**578**	8.333E+006	3.718E+006	2.2	0.008
**580**	3.365E+007	1.696E+007	2.0	0.019
**585**	1.278E+006	2.423E+006	1.9	0.018
**590**	3.154E+006	5.059E+006	1.6	0.010
**591**	2.425E+006	1.096E+006	2.2	0.044
**609**	1.548E+007	1.151E+007	1.3	0.029
**615**	7.314E+005	1.157E+006	1.6	0.017
**617**	1.718E+006	2.899E+006	1.7	0.012
**619**	7.218E+005	1.157E+006	1.6	0.013
**626**	9.726E+005	2.749E+006	1.7	0.012
**628**	5.594E+005	8.948E+005	1.6	0.049
**638**	9.229E+004	2.498E+005	2.7	0.010
**641**	9.151E+005	1.457E+006	1.6	0.011

However, the spots were not visible to naked eye for extraction. After further staining with silver stain, seven spots could be cut from the gel and subsequently subjected to mass spectrometry (Fig 2B). The seven spots were identified as follows: BPI fold-containing family A member 2 precursor (BPIFA2); von Ebner gland protein 1 precursor (VEGP); amylase 1a (2 spots); common salivary protein 1 (CSP1); carbonic anhydrase 6; and cystatin D (Table 2).

**Table 2:** Identification of the protein spots by mass spectrometry.

Spot no.	Protein accession no.	Possible protein	Molecular Weight (Da)	Mascot Protein score	Fold change (in stress group)
517	gi|16258825	BPI fold-containing family A member 2 precursor [Rattus norvegicus]	24685	164	+2.6
585	gi|12621114	von Ebner gland protein 1 precursor [Rattus norvegicus]	19827	164	+1.9
186	gi|56971297	Amy1a protein [Rattus norvegicus]	58149	2950	-1.8
617	gi|392937	Common salivary protein 1 [Rattus norvegicus]	17799	109	+1.7
405	gi|149024685	Carbonic anhydrase 6, isoform CRA_a [Rattus norvegicus]	43947	731	+1.6
641	gi|157818317	Cystatin D precursor [Rattus norvegicus]	17400	156	+1.6
202	gi|56971297	Amy1a protein [Rattus norvegicus]	58149	2831	-1.5

The top two proteins (BPIFA2 and VEGP), based on the fold change, were selected for confirmation using Western blotting. The mean intensities of the BPIFA2 and VEGP bands were significantly higher in the stress group than in the controls (p < 0.05; Fig 2C).

### 3.3 Oral microbiota analysis

**Fig 3:**
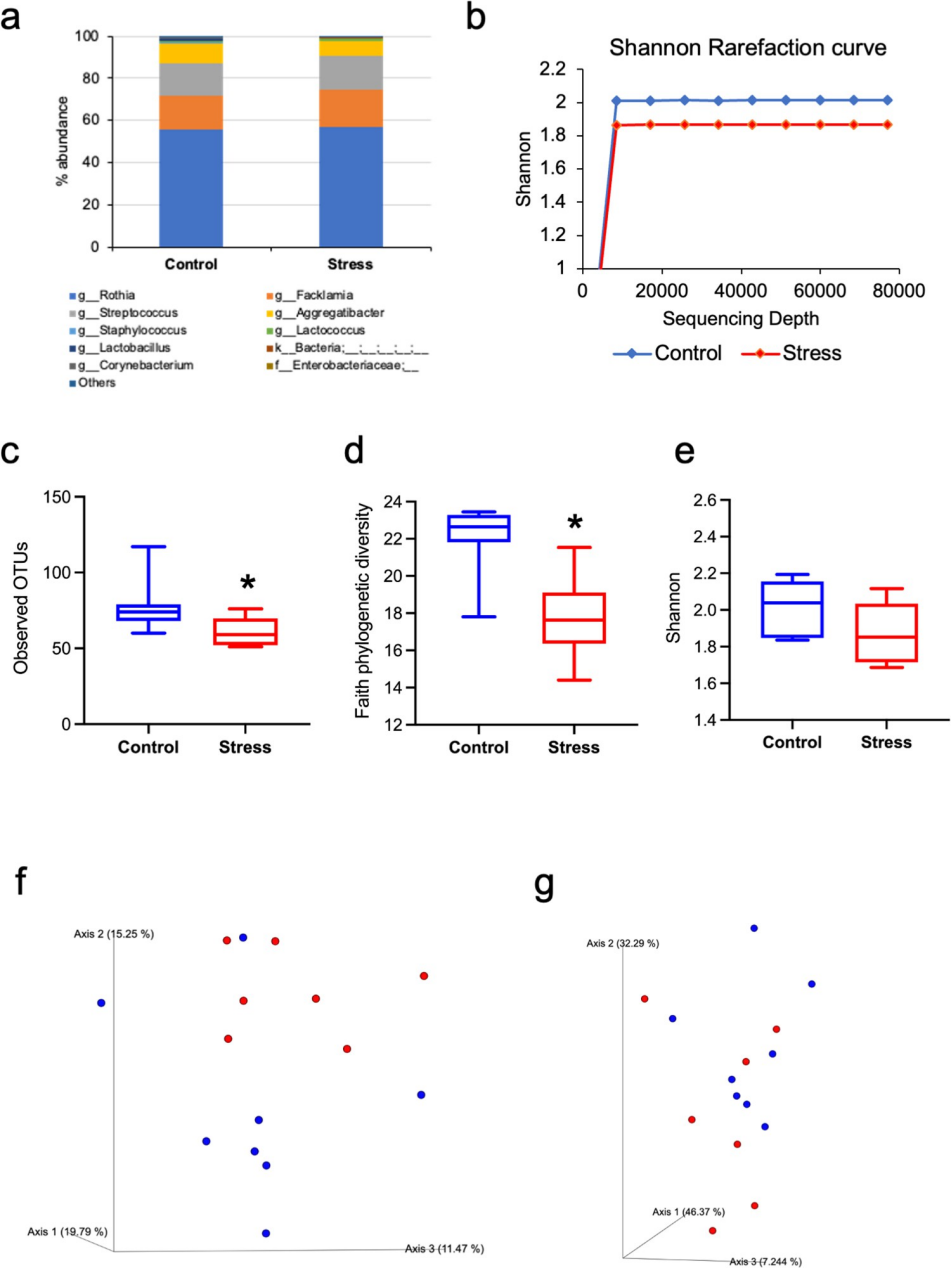
The results of the oral microbiota analysis. (a) The taxonomic abundance shows that oral microbiota was dominated by genus such as *Rothia*, *Facklamia* and *Streptococcus* in both groups. (b) The rarefaction curves plateaued in both the groups indicating sufficient depth of analysis (control group, blue line; stress group, red line). The alpha diversity, as measured by the (c) observed OTUs, (d) faith phylogenetic diversity and (e) Shannon index. The observed OTUs and faith phylogenetic diversity was significantly lower in the stress group compared to that in the control group (*p = 0.02, both; Kruskal-Wallis test). The beta diversity, as measured by the (f) unweighted UniFrac and (g) weighted UniFrac, showed some differences in clustering between the two groups, statistical significance notwithstanding (control group, blue dots; stress group, red dots).

The total sequencing frequency was 1,421,211, and the mean frequency was 94,747 (range, 77,012–115,488). *Rothia*, *Facklamia*, *Streptococcus*, and *Aggregatibacter* were the top 4 genera in the stress and control groups (Fig 3A).

Alpha diversity is a measure of the richness of the species and the evenness within a sample. To adjust for the differences in sequencing depth, rarefaction curve was evaluated which plateaued in both groups indicating a sufficient depth of analysis (Fig 3B). The observed OTUs (p = 0.02; Fig 3B) and faith phylogenetic diversity (p = 0.02, Fig 3D) were significantly lower in the stress group than in the controls.

Beta diversity is a measure of the variation in the microbiota communities between samples. It is assessed using the UniFrac distance matrix and is based on the branch length of the phylogenetic tree shared between the samples. Although some differences in the beta diversity (unweighted UniFrac) of the oral microbiota were observed between the two groups, it was not statistically significant (Fig 3F).

**Fig 4:**
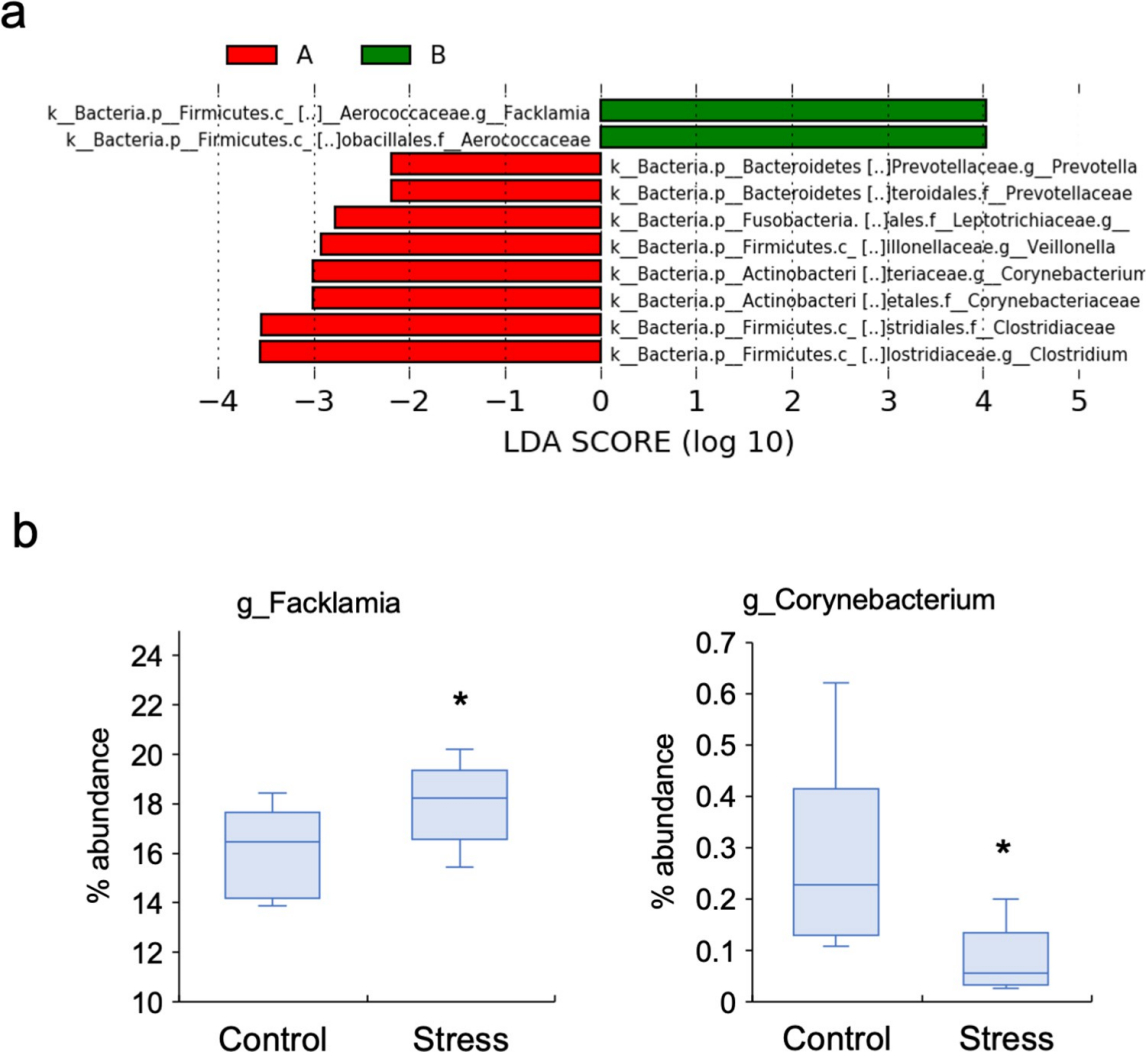
The significantly altered bacteria between stress group and control group. (a) The LEfSe analysis for the LDA score showed alterations in 10 different OTUs between the two groups. (b) The relative abundance of *Facklamia* (at the genus level) was significantly increased, while that of *Corynebacterium* (at the genus level) was significantly decreased in the stress group when compared to those in the control groups (*p < 0.05; Welch’s t-test).

The LEfSe rank plot expressed as LDA logarithmic scores showed 10 differentially abundant bacteria between two groups (Fig 4A). *Facklamia* and *Aerococcaceae* showed higher LDA scores at the genus and family levels, respectively, whereas *Prevotella*, *Veilonella*, *Corynebacterium*, and *Clostridium* showed lower LDA scores at the genus level in the stress group when compared to the control group. Furthermore, analysis of the differences in relative abundances between the two groups using STAMP software showed that the proportion of *Facklamia* was significantly higher (p = 0.03) and that of *Corynebacterium* was significantly lower (p = 0.02) in the stress group when compared to the control group (Fig 4B).

**Fig 5:**
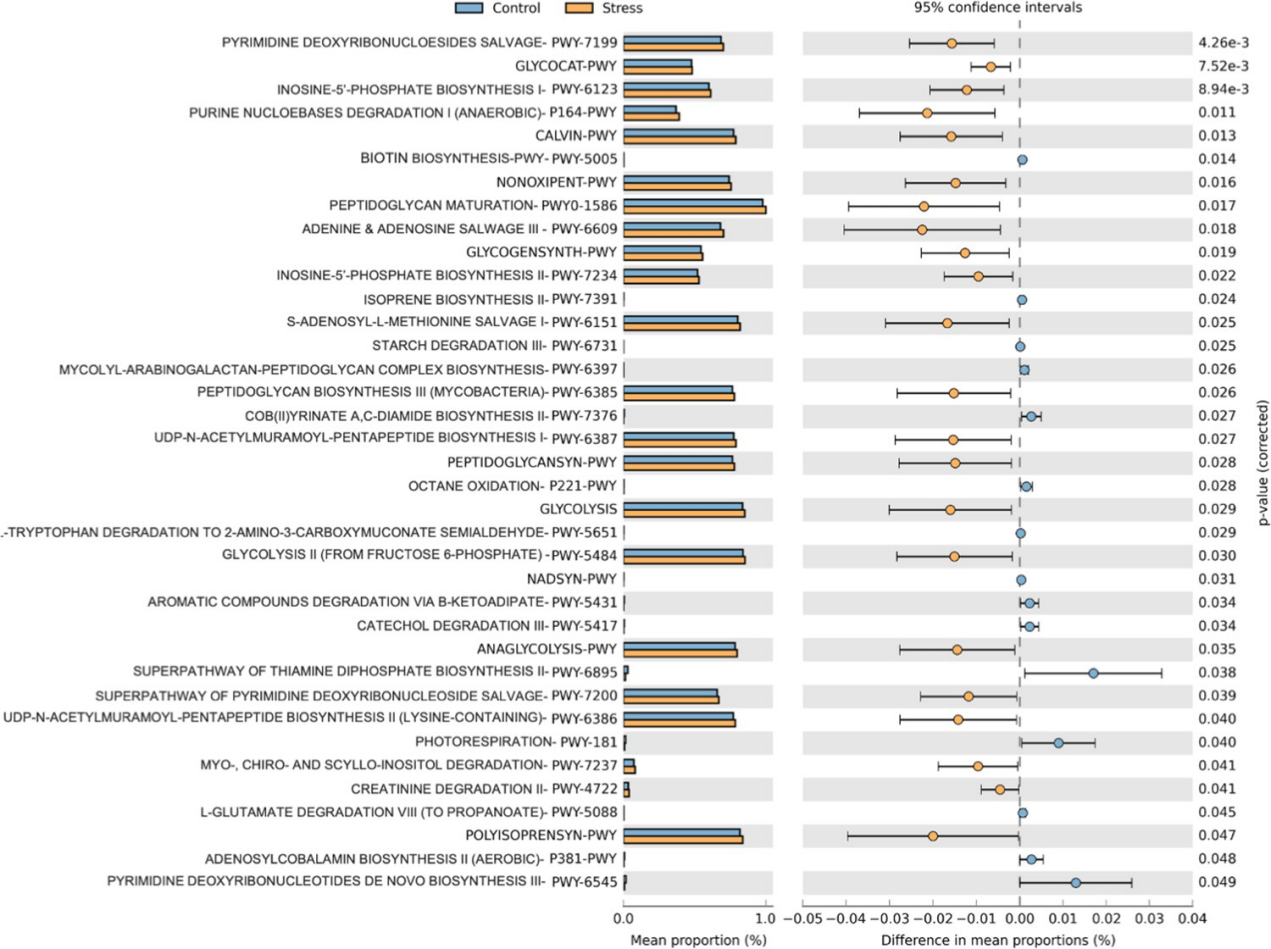
Prediction of metagenome function using PICRUSt2. Thirty-seven different pathways were predicted to alter in the oral microbiota due to psychological stress (*p < 0.05; Welch’s t-tests). Most of the metabolic pathways were upregulated in the stress group indicating high metabolic activity under stress conditions.

STAMP identified 37 significantly altered microbial functional pathways between the two groups, which were predicted using the PICRUSt2 tool (Welch’s t-tests; p < 0.05; Fig 5). Most of these altered metabolic pathways were upregulated in the stress group, suggesting a high metabolic activity of the oral microbiota under psychological stress conditions.

## 4 Discussion

To the best of our knowledge, this is the first study to perform proteomic analysis of the saliva and microbiota analysis of the oral microbiota under psychological stress conditions using an animal model. The proteomic analysis detected significant differences in the expression levels of six salivary proteins between the stress and control groups. These findings were validated by examining the expression levels of two novel proteins, BPIFA2 and VEGP using the Western blot method. The 16S rRNA analysis showed reduced alpha diversity, altered bacterial abundance, and altered predicted metabolic pathways in the oral microbiota under conditions of psychological stress. The identified salivary proteins and the alterations in the oral flora might be involved in stress-related oral diseases.

The stress protocol used in our study was validated using different stress markers, including the body weight, adrenal gland weight, blood corticosterone level, and behavior. The body weight was significantly lower in the stress group from the first week of the experiment until the last week. The weight loss during the initial days of the experiment might be a result of reduced food intake [22], and that after the first week might be due to an increase in the energy expenditure during restraint stress [23]. Increases in both the adrenal gland weight and blood corticosterone level have been used as stress markers [24], and were confirmed in the current study. Previous studies have shown that the increase in the weight of the adrenal gland under stress conditions is due to the hypertrophy of the inner zona fasciculata and hyperplasia of the outer zona fasciculata in the adrenal gland [24]. The blood corticosterone level may rise as a result of the hyperactivity of the HPA axis. Behavior analysis in rodents is an important aspect of stress-related studies. Several types of behavior analyses, such as the EPM test, open field test, forced swim test, novelty exploration, and tail suspension have been used to determine whether the animal is under stress [25]. The EPM test is widely used because of its simple design, ease of use, and quantifiable behavior responses [18]. This test has been clinically validated as an indicator of human anxiety [26]. The avoidance of the open arm, perceived as a potential danger, is characteristic of stressed rodents. The lower number of entries and time spent in the open arm by the rats in the stress group in the current study suggested that they were under high levels of stress compared to the controls.

In the present study, the two most differentially expressed proteins, BPIFA2 and VEGP were confirmed to be elevated in the stress group by Western blot analyses. BPIFA2 or Parotid Secretory Protein is a palate, lung, and nasal epithelial clone family protein and is expressed in human salivary glands, constituting ~0.5% of the total proteins in human saliva [27]. BPIFA2 has been shown to function as a salivary surfactant; the saliva from a BPIFA2 knock-out mouse demonstrated diminished ability to spread on the surface when compared to that from a wild-type mouse [28]. The increase in the level of BPIFA2 under psychological stress indicates that stress might increase the surfactant property of saliva. Additionally, BPIFA2 promotes the colonization of *Candida albicans* to the silicone prosthesis [29], which might explain the increase in *Candida* species in stress-related oral diseases [30,31]. However, additional studies are needed to clarify these speculations.

VEGP/Lipocalin-1 is secreted by the von Ebner’s gland which is located around the circumvallate and foliate papilla in the tongue. It plays a role in the perception of bitter taste; bitter compounds bind to this protein, which is then transported to the circumvallate and foliate papilla for the perception of taste [32]. The increase in the level of VEGP under psychological stress indicates that psychological stress can affect the sensation of taste. Patients with burning mouth syndrome, a stress-related oral symptom, often complain of taste alterations, which might be consistent with an increase in the level of VEGP. Further studies are required to clarify this phenomenon.

The other proteins identified via mass spectrometry in the current study have been shown to play roles in oral and systemic diseases. CSP1 was increased in the saliva of patients with periodontitis, pancreatic cancer, and diabetes implying its involvement in both oral and systemic diseases [33–35]. Carbonic anhydrase 6 is responsible for the physiologic processes in the oral cavity, such as pH regulation and carbon dioxide and bicarbonate transport through the buffer system [36]. High activity of carbonic anhydrase 6 in the saliva might induce biofilm formation and aid in the development of dental caries [37]. The high expression level of carbonic anhydrase 6 may affect dental caries activity under stress conditions. In addition, carbonic anhydrase 6 is involved in bitter taste perception [38]. Taste disturbances are often induced by psychological stress [39,40]; hence, this protein might play a role in stress-related disturbances in taste. Cystatin D is a cysteine protease inhibitor [41]. Although the effect of psychological stress on cystatin D has not been demonstrated so far, a decrease in the level of this protein in saliva was observed in a group of central nerve disorders, including neurodevelopmental and autism spectrum disorders [42]. The salivary level of cystatin D might be affected by the function of the central nervous system. Further experiments are needed to clarify this speculation.

The expression of alpha-amylase 1 was reduced in the saliva of the stressed rats when compared to that of the controls (Table 2). An increase in the level of alpha-amylase has been used as a salivary stress marker for acute stress [43]. Alternatively, a decrease in the level of this protein in chronic stress has been reported [44,45]. Salivary alpha-amylase rapidly increases during stress conditions and returns to baseline immediately after the stress is removed [45]. Acute and chronic stress conditions may increase or decrease the production of alpha-amylase, respectively. Hence, reduced levels of salivary amylase may be further studied for use as another marker of chronic stress.

A limitation to proteomic analysis in our study is that we used a conventional gel-based approach to identify the altered protein. The silver staining method for gel may have missed the staining of more significantly altered protein spots. The use of other advanced and sensitive methods such as mass spectrometry coupled with shotgun proteomics could be useful in identifying the altered proteins more precisely.

The microbiota analysis showed reduced alpha diversity, altered bacterial abundance, and predicted metabolic pathways in the oral microbiota under stress conditions. The alpha diversity of the oral microbiota was significantly reduced in the stress group compared to the controls. Reduced alpha diversity has been observed in patients with RAS and oral lichen planus [46]. Our findings may be consistent with the concept that psychological stress is an etiology of RAS and oral lichen planus [2]. A reduction in the alpha diversity of the oral microbiota was observed in patients with oral mucositis after radiotherapy [47] thus indicating that reduced alpha diversity due to psychological stress might be considered as a risk factor for oral mucositis.

The proportions of *Facklamia* and *Corynebacterium* were increased and decreased, respectively in the stress group, as shown by both the LefSe and STAMP software analyses in this study. *Facklamia* is a gram-positive, α-hemolytic, facultative anaerobic bacterium commonly associated with endocarditis and bacteremia [48]. In a rat depression model, the abundance of *Facklamia* was decreased in the gut after treatment with Paeoniflorin, a Chinese herb known for its antidepressant property [49]. Although studies on the association between *Facklamia* and psychological disorders are limited, the findings of the current study and the aforementioned report imply a relationship between the two factors. Nonetheless, further studies are required to clarify this speculation. *Corynebacterium* is a gram-positive bacterium that directly synthesizes serotonin, a stress response neurotransmitter in the gut [50]. Lower levels of *Corynebacterium* were observed in the guts of the depressed rat models [51]. The decrease in the abundance of *Corynebacterium* in the oral cavity during psychological stress in the current study might be related to the stress response. Alterations in the abundance of *Facklamia* and *Corynebacterium* have been reported only in the guts of stress models; to the best of our knowledge, the current study is the first to report these findings in the oral cavity.

A total of 37 different pathways were predicted to be altered between stress and control groups in this study. Most of the altered pathways were related to nucleotide, glucose, vitamin, carbohydrate, and amino acids biosynthesis and degradation. The stress group showed significant upregulations in these metabolic pathways suggesting that the metabolic activity is higher under psychological stress. Although the finding is insufficient to be directly correlated with the clinical implications, this study provides evidence that psychological stress has a significant impact on the oral microbiota, including the metabolic pathways.

## 5 Conclusion

The present study identified salivary proteins that were altered under psychological stress in a rat restraint stress model. In addition, this study provided evidence that psychological stress can cause significant alterations in the oral microbiota. These findings might aid in understanding the pathogenesis of stress-related oral diseases.

## Supporting information

S1 Fig Stress method and behavior analyses. (a) The stress group rats were enclosed in a plastic tube with ventilation for 4 hours daily over a period of 1 month. (b) After a month of stress, the behavior of rats was analyzed using elevated plus maze test. The rats were placed at the center of the maze facing the open arm and allowed to explore it for 10 min. The number of entries and the time spent in the open and closed arms by each rat were recorded manually.
(TIF)

S2 Fig Analysis of intestinal microbiota. (a) Taxonomic abundance of intestinal microbiota. (b) The LefSe analysis showed reduced abundance of bacteria such as *Lactobacillus* and *Bifidobacterium* in stress group.
(TIF)

S3 Fig The beta diversity of intestinal microbiota. The weighted (a) and unweighted UniFrac (b) also showed significant differences between stress and control group (Blue dots- Control; Red dots- Stress).
(TIF)

S4 Fig The gel images used for quantification of protein spots is deposited in figshare and is available as https://doi.org/10.6084/m9.figshare.19333859.v1. (PDF)

S1 Raw images Blot images of western blot. The blot images are deposited in figshare and is available as https://doi.org/10.6084/m9.figshare.19387415.v1.
(PDF)

## References

[1] Yang C, Liu L, Shi H, Zhang Y. Psychological problems and quality of life of patients with oral mucosal diseases: a preliminary study in Chinese population. BMC Oral Health. 2018; 18:226. doi: 10.1186/s12903-018-0696-y 30587180 PMC6307175

[2] Abiko Y, Paudel D, Matsuoka H, Yamazaki Y, Koga C, Kitagawa Y, et al. Psychostomatology: The psychosomatic status and approaches for the management of patients with inflammatory oral mucosal diseases. J Oral Maxillofac Surgery, Med Pathol. 2021; doi: 10.1016/j.ajoms.2021.08.007

[3] Martina E, Campanati A, Diotallevi F, Offidani A. Saliva and Oral Diseases. J Clin Med. 2020; 9(2):466. doi: 10.3390/jcm9020466 32046271 PMC7074457

[4] Soo-Quee Koh D, Choon-Huat Koh G. The use of salivary biomarkers in occupational and environmental medicine. Occup Environ Med. 2007; 64(3):202–210. doi: 10.1136/oem.2006.026567 17339296 PMC2092532

[5] Obayashi K. Salivary mental stress proteins. Clin Chim Acta. 2013; 425:196–201. doi: 10.1016/j.cca.2013.07.028 23939251

[6] Eguia A, Martinez-Conde R, Lopez J, Uribarri A, Aguirre J. Salivary levels of Tumour Necrosis Factor-alpha in patients with recurrent aphthous stomatitis. Med Oral Patol Oral y Cir Bucal. 2011; 16(1):e33–e36.

[7] Humberto JSM, Pavanin JV, Rocha MJA da, Motta ACF. Cytokines, cortisol, and nitric oxide as salivary biomarkers in oral lichen planus: a systematic review. Braz Oral Res. 2018; 32:e82. doi: 10.1590/1807-3107bor-2018.vol32.0082 30110084

[8] Kishore J, Shaikh F, Mirza S, Raffat MA, Ikram S, Akram Z. Cytokine levels and their role in the etiopathogenesis of Burning Mouth Syndrome: A systematic review. Cephalalgia. 2019; 39(12):1586–1594. doi: 10.1177/0333102419854052 31132870

[9] Wade WG. The oral microbiome in health and disease. Pharmacol Res. 2013; 69(1):137–143. doi: 10.1016/j.phrs.2012.11.006 23201354

[10] Graves DT, Corrêa JD, Silva TA. The Oral Microbiota Is Modified by Systemic Diseases. J Dent Res. 2019; 98(2):148–156. doi: 10.1177/0022034518805739 30359170 PMC6761737

[11] Yoshizawa JM, Schafer CA, Schafer JJ, Farrell JJ, Paster BJ, Wong DT. Salivary biomarkers: toward future clinical and diagnostic utilities. Clin Microbiol Rev. 2013;26(4):781–91. doi: 10.1128/CMR.00021-13 24092855 PMC3811231

[12] Farrell JJ ZL, Zhou H, Chia D, Elashoff D, Akin D, Paster BJ, et al. Variations of oral microbiota are associated with pancreatic diseases including pancreatic cancer. Gut. 2012;61:582–588. doi: 10.1136/gutjnl-2011-300784 21994333 PMC3705763

[13] Mager D, Haffajee A, Devlin P, Norris C, Posner M, Goodson J. 2005. The salivary microbiota as a diagnostic indicator of oral cancer: a descriptive, non-randomized study of cancer-free and oral squamous cell carcinoma subjects. J. Transl. Med. 2012;3:27.

[14] Duran-Pinedo AE, Solbiati J, Frias-Lopez J. The effect of the stress hormone cortisol on the metatranscriptome of the oral microbiome. npj Biofilms and Microbiomes. 2018;4:25. doi: 10.1038/s41522-018-0068-z 30345066 PMC6194028

[15] Festing MFW. On determining sample size in experiments involving Laboratory Animals. Laboratory Animals. 2018;52: 341–350. doi: 10.1177/0023677217738268 29310487

[16] Yun J, Koike H, Ibi D, Toth E, Mizoguchi H, Nitta A, et al. Chronic restraint stress impairs neurogenesis and hippocampus-dependent fear memory in mice: possible involvement of a brain-specific transcription factor Npas4. J Neurochem. 2010; 114(6):1840–1851. doi: 10.1111/j.1471-4159.2010.06893.x 20626564

[17] Abusleme L, Hong B-Y, Hoare A, Konkel J, Diaz P, Moutsopoulos N. Oral Microbiome Characterization in Murine Models. Bio Protoc. 2017; 7(24):e2655. doi: 10.21769/BioProtoc.2655 29333479 PMC5760993

[18] Walf AA, Frye CA. The use of the elevated plus maze as an assay of anxiety-related behavior in rodents. Nat Protoc. 2007; 2(2):322–328. doi: 10.1038/nprot.2007.44 17406592 PMC3623971

[19] Karl JP, Hatch AM, Arcidiacono SM, Pearce SC, Pantoja-Feliciano IG, Doherty LA, et al. Effects of Psychological, Environmental and Physical Stressors on the Gut Microbiota. Front Microbiol. 2018; 9:2013. doi: 10.3389/fmicb.2018.02013 30258412 PMC6143810

[20] Fourie NH, Wang D, Abey SK, et al. Structural and functional alterations in the colonic microbiome of the rat in a model of stress induced irritable bowel syndrome. Gut Microbes. 2017;8(1):33–45. doi: 10.1080/19490976.2016.1273999 28059627 PMC5341915

[21] Xu M, Wang C, Krolick KN, Shi H, Zhu J. Difference in post-stress recovery of the gut microbiome and its altered metabolism after chronic adolescent stress in rats. Sci Rep. 2020;10(1):3950. doi: 10.1038/s41598-020-60862-1 32127581 PMC7054252

[22] Jeong JY, Lee DH, Kang SS. Effects of chronic restraint stress on body weight, food intake, and hypothalamic gene expressions in mice. Endocrinology and Metabolism. 2013;28: 288–296. doi: 10.3803/EnM.2013.28.4.288 24396694 PMC3871039

[23] Bhatnagar S, Vining C, Iyer V, Kinni V. Changes in hypothalamic-pituitary-adrenal function, body temperature, body weight and food intake with repeated social stress exposure in rats. Journal of Neuroendocrinology. 2006;18: 13–24. doi: 10.1111/j.1365-2826.2005.01375.x 16451216

[24] Ulrich-Lai YM, Figueiredo HF, Ostrander MM, Choi DC, Engeland WC, Herman JP. Chronic stress induces adrenal hyperplasia and hypertrophy in a subregion-specific manner. American Journal of Physiology-Endocrinology and Metabolism. 2006;291(5):E965–73. doi: 10.1152/ajpendo.00070.2006 16772325

[25] Belovicova K, Bogi E, Csatlosova K, Dubovicky M. Animal tests for anxiety-like and depression-like behavior in rats. Interdisciplinary Toxicology. 2017;10: 40–43. doi: 10.1515/intox-2017-0006 30123035 PMC6096862

[26] Biedermann SV, Biedermann DG, Wenzlaff F, Kurjak T, Nouri S, Auer MK, et al. An elevated plus-maze in mixed reality for studying human anxiety-related behavior. BMC Biology. 2017;15:125. doi: 10.1186/s12915-017-0463-6 29268740 PMC5740602

[27] Prokopovic V, Popovic M, Andjelkovic U, Marsavelski A, Raskovic B, Gavrovic-Jankulovic M, et al. Isolation, biochemical characterization and anti-bacterial activity of BPIFA2 protein. Arch Oral Biol. 2014;59(3):302–9. doi: 10.1016/j.archoralbio.2013.12.005 24581853

[28] Nandula SR, Huxford I, Wheeler TT, Aparicio C, Gorr S. The parotid secretory protein BPIFA2 is a salivary surfactant that affects lipopolysaccharide action. Exp Physiol. 2020. 105(8):1280–1292. doi: 10.1113/EP088567 32390232 PMC9484039

[29] Holmes AR, Rodrigues E, van der Wielen P, Lyons KM, Haigh BJ, Wheeler TT, et al. Adherence of Candida albicans to silicone is promoted by the human salivary protein SPLUNC2/PSP/BPIFA2. Mol Oral Microbiol. 2014; 29(2):90–98. doi: 10.1111/omi.12048 24506943

[30] Hatchuel DA, Peters E, Lemmer J, Hille JJ, McGaw WT. Candidal infection in oral lichen planus. Oral Surgery, Oral Med Oral Pathol. 1990;70(2):172–175. doi: 10.1016/0030-4220(90)90113-7 2290645

[31] Núñez MJ, Balboa J, Riveiro P, Liñares D, Mañá P, Rey-Méndez M, et al. Effects of Psychological Stress and Alprazolam on Development of Oral Candidiasis in Rats. Clin Vaccine Immunol. 2002;9(4):852–857. doi: 10.1128/cdli.9.4.852-857.2002 12093685 PMC120028

[32] Flower DR. The lipocalin protein family: structure and function. Biochem J. 1996; 318(1):1–14. doi: 10.1042/bj3180001 8761444 PMC1217580

[33] Kim SA, Lee Y, Jung DE, Park KH, Park JY, Gang J, et al. Pancreatic adenocarcinoma up-regulated factor (PAUF), a novel up-regulated secretory protein in pancreatic ductal adenocarcinoma. Cancer Sci. 2009;100(5):828–836. doi: 10.1111/j.1349-7006.2009.01106.x 19302292 PMC11159838

[34] Heo S-M, Lee S, Wang H, Jeong JH, Oh SW. Levels of common salivary protein 1 in healthy subjects and periodontal patients. J Periodontal Implant Sci. 2016;46(5):320–328. doi: 10.5051/jpis.2016.46.5.320 27800214 PMC5083815

[35] Wang H, Heo S-M, Jin HY, Choi EY, Oh SW. Common Salivary Protein 1 in Serum of Diabetes Patients. J Clin Lab Anal. 2016;30(6):961–967. doi: 10.1002/jcla.21963 27076118 PMC6807161

[36] Parkkila S, Kaunisto K, Rajaniemi L, Kumpulainen T, Jokinen K, Rajaniemi H. Immunohistochemical localization of carbonic anhydrase isoenzymes VI, II, and I in human parotid and submandibular glands. J Histochem Cytochem. 1990;38(7):941–947. doi: 10.1177/38.7.2113069 2113069

[37] Picco DCR, Marangoni-Lopes L, Parisotto TM, Mattos-Graner R, Nobre-dos-Santos M. Activity of Carbonic Anhydrase VI is Higher in Dental Biofilm of Children with Caries. Int J Mol Sci. 2019;20(11):2673. doi: 10.3390/ijms20112673 31151296 PMC6600353

[38] Patrikainen M, Pan P, Kulesskaya N, Voikar V, Parkkila S. The role of carbonic anhydrase VI in bitter taste perception: evidence from the Car6^−/−^ mouse model. J Biomed Sci. 2014;21:82. doi: 10.1186/s12929-014-0082-2 25134447 PMC4237775

[39] Bergdahl M, Bergdahl J. Perceived taste disturbance in adults: prevalence and association with oral and psychological factors and medication. Clin Oral Investig. 2002;6(3):145–149. doi: 10.1007/s00784-002-0169-0 12271346

[40] Heath TP, Melichar JK, Nutt DJ, Donaldson LF. Human Taste Thresholds Are Modulated by Serotonin and Noradrenaline. J Neurosci. 2006;26(49):12664–12671. doi: 10.1523/JNEUROSCI.3459-06.2006 17151269 PMC6674841

[41] Hunaiti S, Wallin H, Eriksson M, Järås M, Abrahamson M. Secreted cystatins decrease proliferation and enhance apoptosis of human leukemic cells. FEBS Open Bio. 2020; 10(10):2166–2181. doi: 10.1002/2211-5463.12958 32810913 PMC7530398

[42] Ngounou Wetie AG, Wormwood KL, Charette L, Ryan JP, Woods AG, Darie CC. Comparative two‐dimensional polyacrylamide gel electrophoresis of the salivary proteome of children with autism spectrum disorder. J Cell Mol Med. 2015;19(11):2664–2678. doi: 10.1111/jcmm.12658 26290361 PMC4627571

[43] Akiyoshi J, Tanaka Y, Isogawa K, Ishitobi Y, Tsuru J, Ando T, et al. Acute Stress in Patients with Panic Disorder Produces Effects on Salivary Amylase and Cortisol. In: Selek S, editor. Different Views of Anxiety Disorders. London (UK): IntechOpen Limited. 2011. doi: 10.5772/18556

[44] Wolf JM, Nicholls E, Chen E. Chronic stress, salivary cortisol, and α-amylase in children with asthma and healthy children. Biol Psychol. 2008;78(1):20–28. doi: 10.1016/j.biopsycho.2007.12.004 18243483

[45] Maruyama Y, Kawano A, Okamoto S, Ando T, Ishitobi Y, Tanaka Y, et al. Differences in Salivary Alpha-Amylase and Cortisol Responsiveness following Exposure to Electrical Stimulation versus the Trier Social Stress Tests. PLoS One. 2012;7(7):e39375. doi: 10.1371/journal.pone.0039375 22859941 PMC3408464

[46] Hijazi K, Morrison RW, Mukhopadhya I, Martin B, Gemmell M, Shaw S, et al. Oral bacterial diversity is inversely correlated with mucosal inflammation. Oral Dis. 2020;26(7):1566–1575. doi: 10.1111/odi.13420 32419230

[47] Zhu X-X, Yang X-J, Chao Y-L, Zheng H-M, Sheng H-F, Liu H-Y, et al. The Potential Effect of Oral Microbiota in the Prediction of Mucositis During Radiotherapy for Nasopharyngeal Carcinoma. EBioMedicine. 2017;18:23–31. doi: 10.1016/j.ebiom.2017.02.002 28216066 PMC5405060

[48] Rahmati E, Martin V, Wong D, Sattler F, Petterson J, Ward P, et al. Facklamia Species as an Underrecognized Pathogen. Open Forum Infect Dis. 2017;4(1): ofw272. doi: 10.1093/ofid/ofw272 28480264 PMC5414014

[49] Yu J-B, Zhao Z-X, Peng R, Pan L-B, Fu J, Ma S-R, et al. Gut Microbiota-Based Pharmacokinetics and the Antidepressant Mechanism of Paeoniflorin. Front Pharmacol. 2019;10:268. doi: 10.3389/fphar.2019.00268 30949054 PMC6435784

[50] Kanova M, Kohout P. Serotonin—Its Synthesis and Roles in the Healthy and the Critically Ill. Int J Mol Sci. 2021;22(9):4837. doi: 10.3390/ijms22094837 34063611 PMC8124334

[51] Yu M, Jia H, Zhou C, Yang Y, Zhao Y, Yang M, et al. Variations in gut microbiota and fecal metabolic phenotype associated with depression by 16S rRNA gene sequencing and LC/MS-based metabolomics. J Pharm Biomed Anal. 2017;138:231–239. doi: 10.1016/j.jpba.2017.02.008 28219800

