# Supplementary figures and images for: Proteomic and microbiota analyses of the oral cavity during psychological stress
Source: PLoS One. 2022 May 25;17(5):e0268155. doi: 10.1371/journal.pone.0268155 (PMC9132284; doi:10.1371/journal.pone.0268155)

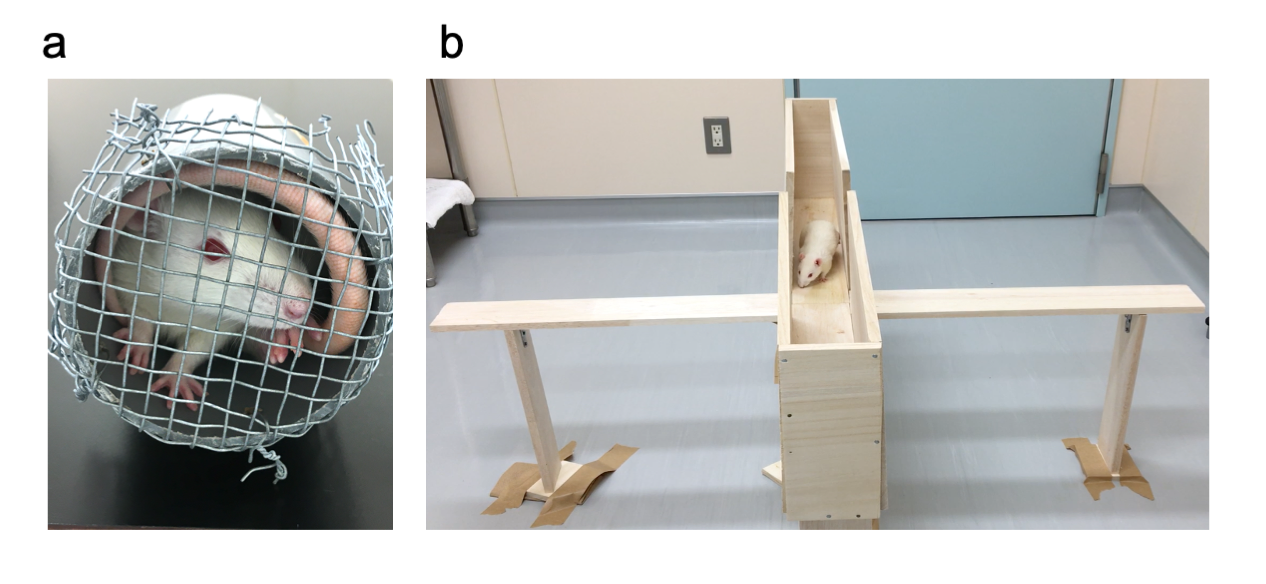

Supplement: S1 Fig — (a) The stress group rats were enclosed in a plastic tube with ventilation for 4 hours daily over a period of 1 month. (b) After a month of stress, the behavior of rats was analyzed using elevated plus maze test. The rats were placed at the center of the maze facing the open arm and allowed to explore it for 10 min. The number of entries and the time spent in the open and closed arms by each rat were recorded manually. (TIF) [file pone.0268155.s001.tif]

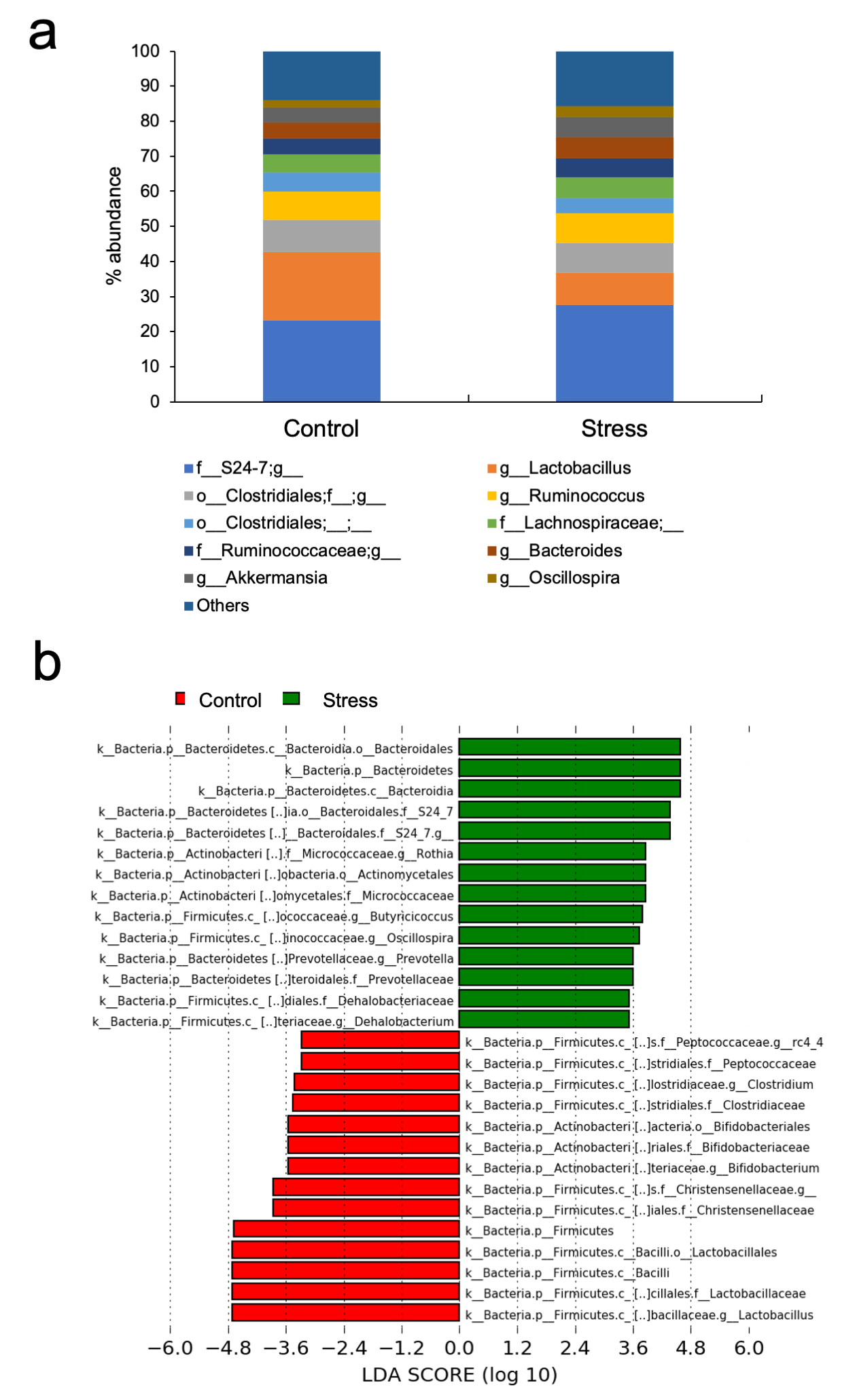

Supplement: S2 Fig — (a) Taxonomic abundance of intestinal microbiota. (b) The LefSe analysis showed reduced abundance of bacteria such as Lactobacillus and Bifidobacterium in stress group. (TIF) [file pone.0268155.s002.tif]

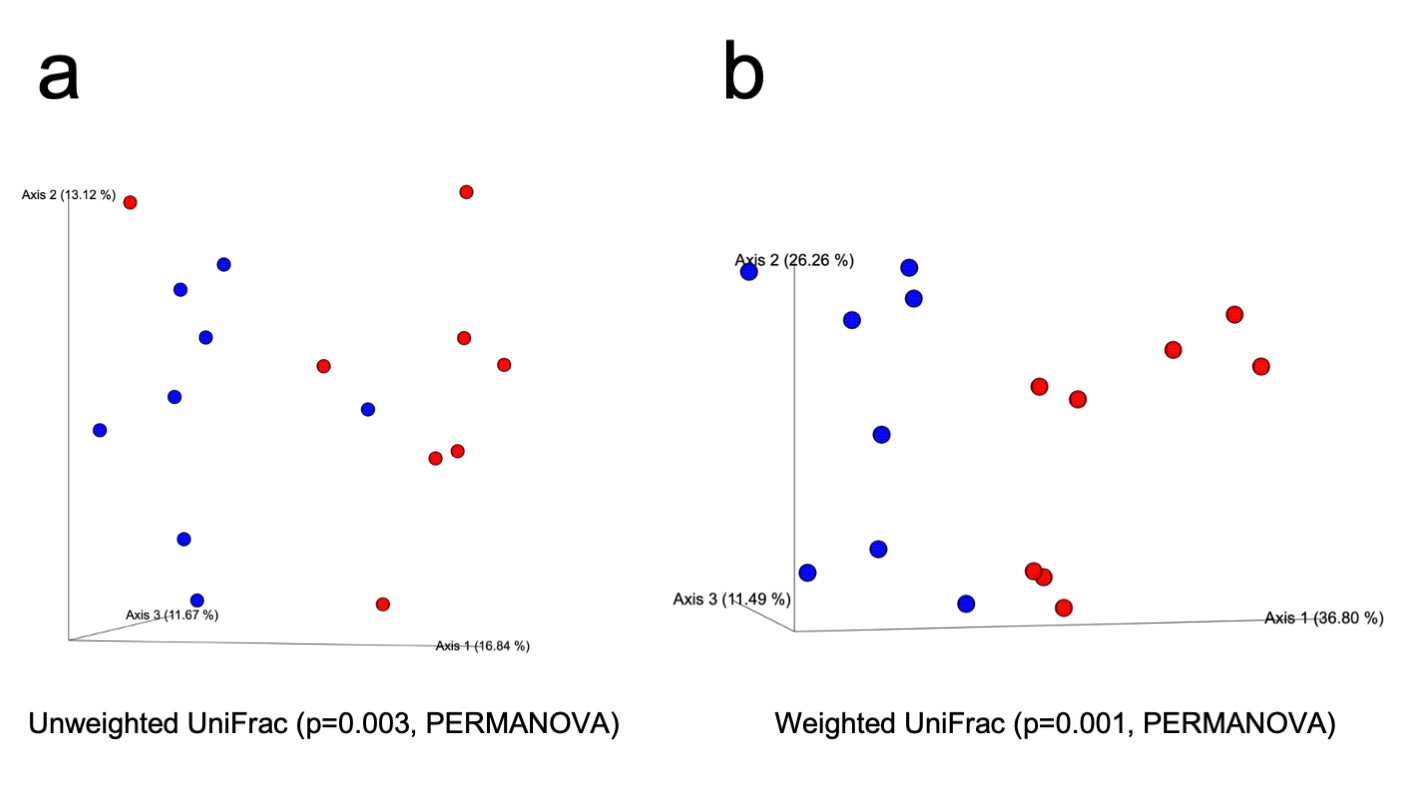

Supplement: S3 Fig — The weighted (a) and unweighted UniFrac (b) also showed significant differences between stress and control group (Blue dots- Control; Red dots- Stress). (TIF) [file pone.0268155.s003.tif]
