# Supplementary material for: Proteomic and microbiota analyses of the oral cavity during psychological stress
Source: PLoS One. 2022 May 25;17(5):e0268155. doi: 10.1371/journal.pone.0268155 (PMC9132284; doi:10.1371/journal.pone.0268155)
Supplement: S4 Fig — (PDF) [file pone.0268155.s004.pdf]

Gel images after fluorescent staining used for quantification

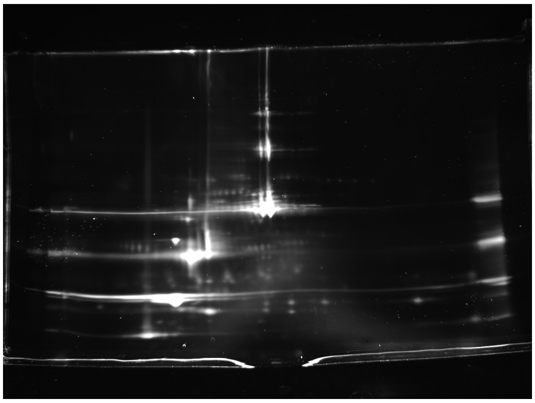

Control 1

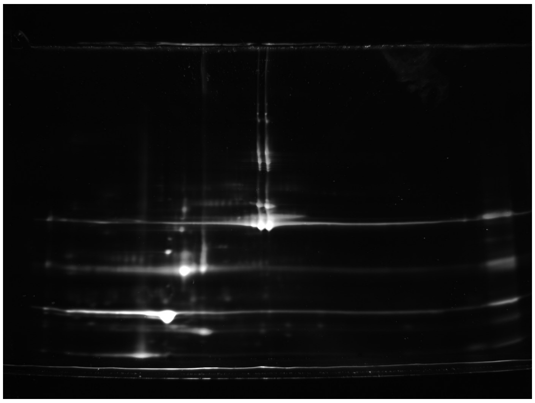

Control 2

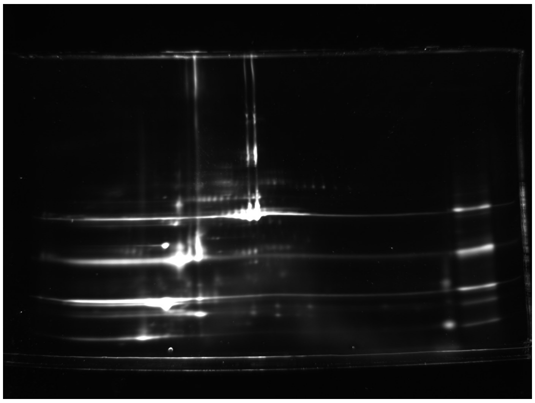

Control 3

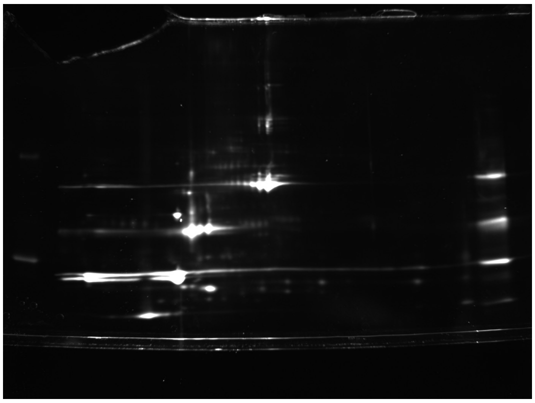

Control 4

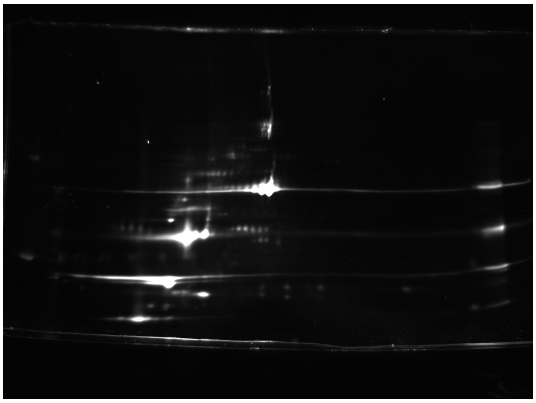

Control 5

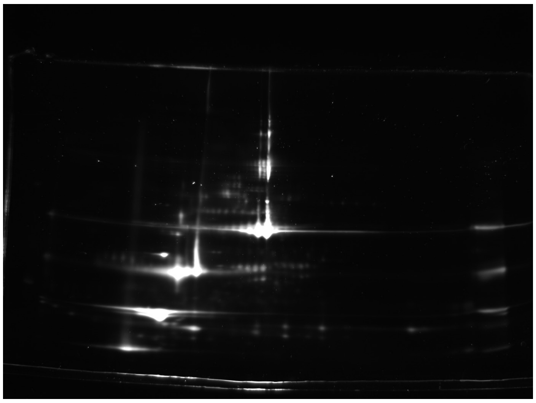

Control 6

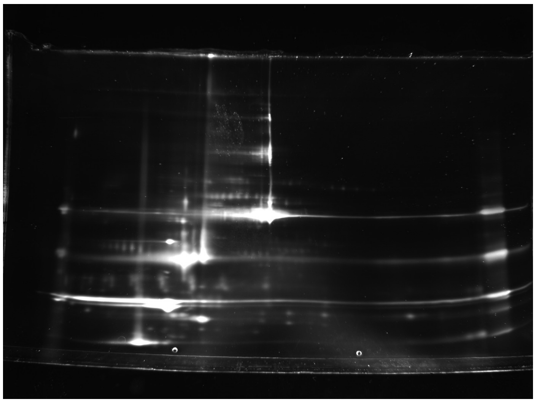

Control 7

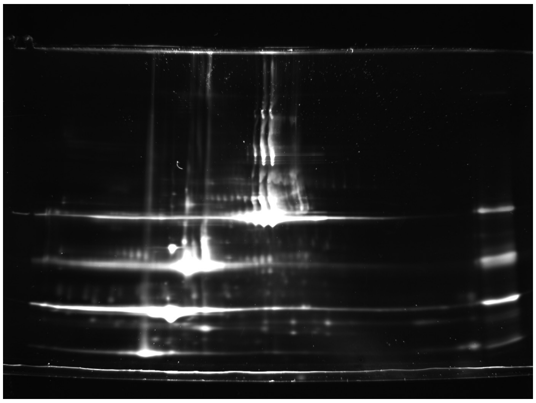

Control 8

Gel images after fluorescent staining used for quantification

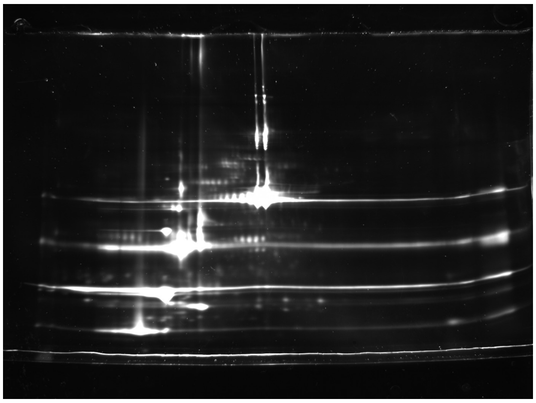

Stress 1

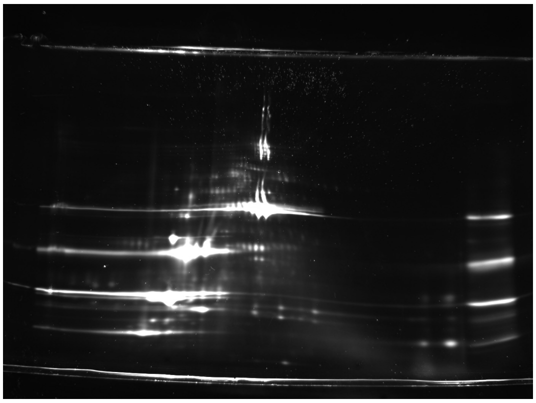

Stress 2

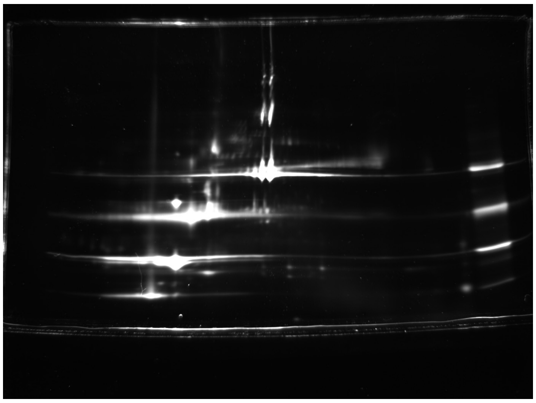

Stress 3

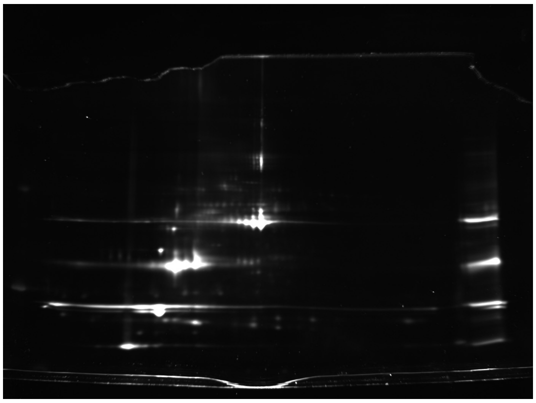

Stress 4

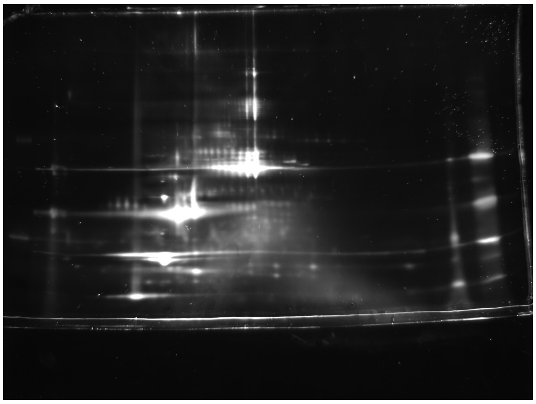

Stress 5

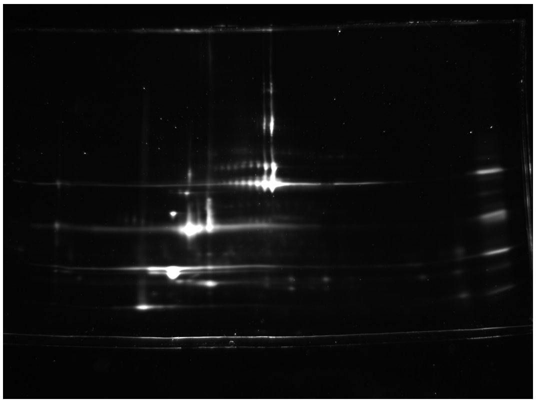

Stress 6

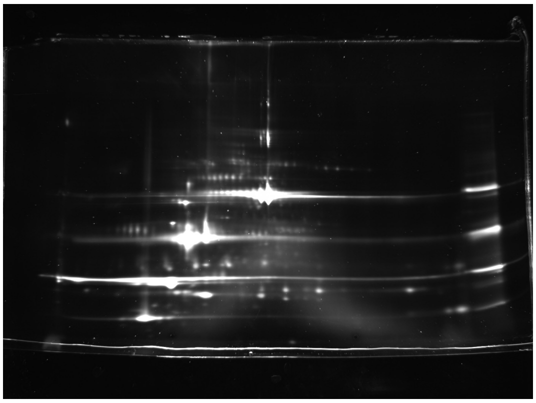

Stress 7

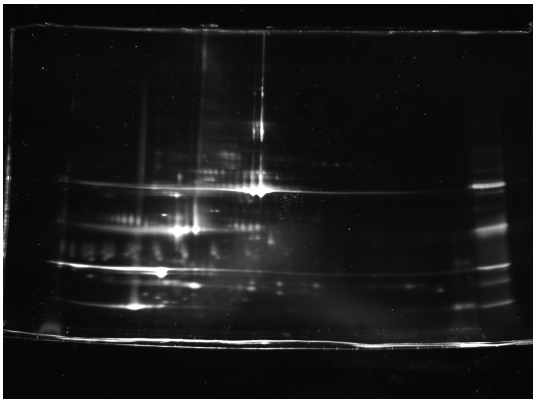

Stress 8
