# Supplementary material for: Proteomic and microbiota analyses of the oral cavity during psychological stress
Source: PLoS One. 2022 May 25;17(5):e0268155. doi: 10.1371/journal.pone.0268155 (PMC9132284; doi:10.1371/journal.pone.0268155)
Supplement: S1 Raw images — The blot images are deposited in figshare and is available as https://doi.org/10.6084/m9.figshare.19387415.v1. (PDF) [file pone.0268155.s005.pdf]

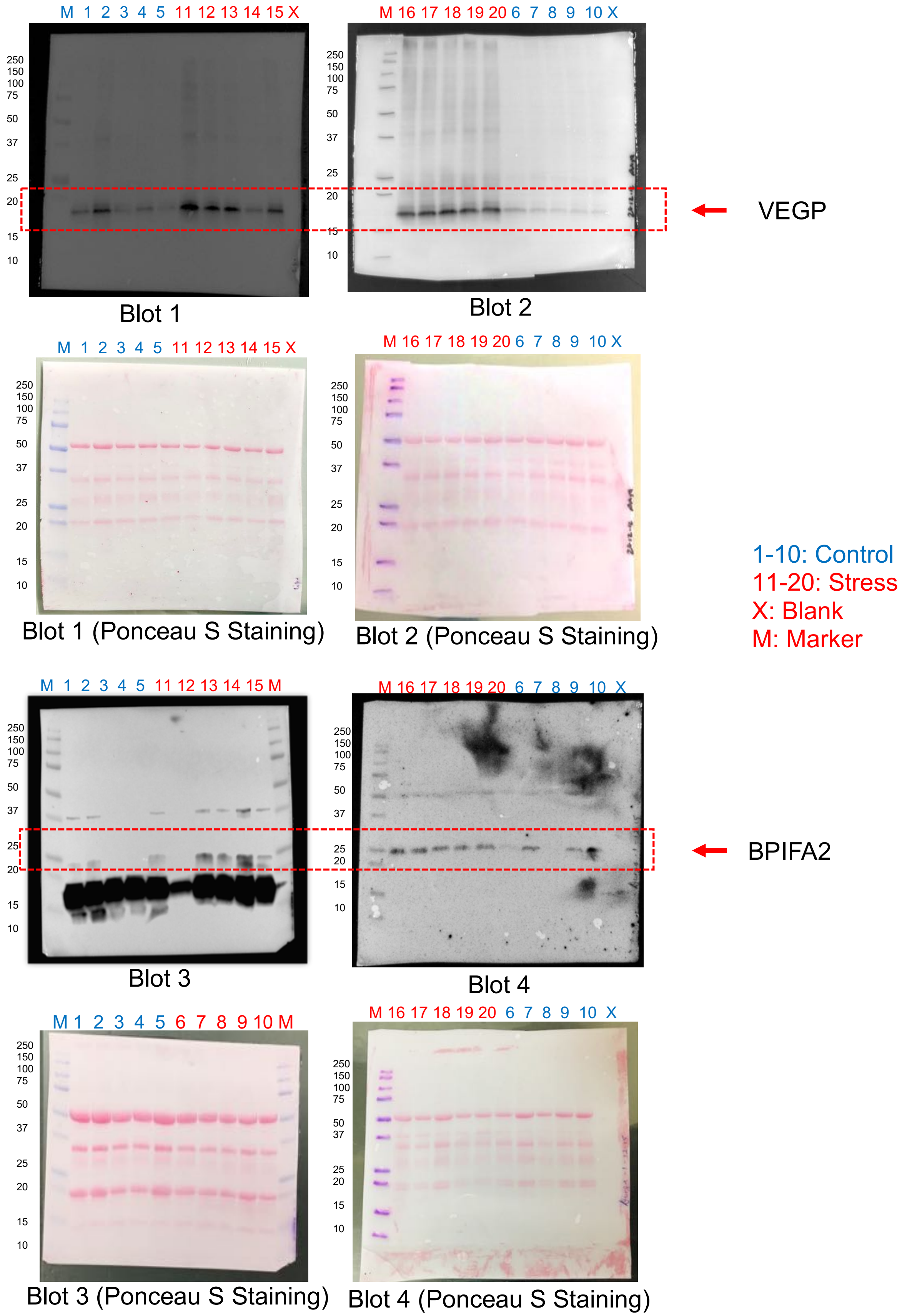
M 1 2 3 4 5 11 12 13 14 15 X
M 16 17 18 19 20 6 7 8 9 10 X
250
150
100
75
50
37
25
20
15
10
VEGP
Blot 1
Blot 2
Blot 1 (Ponceau S Staining)
Blot 2 (Ponceau S Staining)
1-10: Control
11-20: Stress
X: Blank
M: Marker
M 1 2 3 4 5 11 12 13 14 15 M
BPIFA2
Blot 3
Blot 4
M 1 2 3 4 5 6 7 8 9 10 M
Blot 3 (Ponceau S Staining)
Blot 4 (Ponceau S Staining)
